# Barriers and facilitators for sustainable weight loss in the pre-conception period among Danish women with overweight or obesity – a qualitative study

**DOI:** 10.1186/s12889-023-16676-7

**Published:** 2023-09-13

**Authors:** Majken Lillholm Pico, Louise Groth Grunnet, Christina Anne Vinter, Jens Aagaard-Hansen, Karoline Kragelund Nielsen

**Affiliations:** 1 Health Promotion Research, Steno Diabetes Center Copenhagen, 2730 Herlev, Denmark; 2 Clinical Research, Steno Diabetes Center Copenhagen, 2730 Herlev, Denmark; 3 Department of Gynecology and Obstetrics and Steno Diabetes Center Odense, Odense University Hospital, Odense, Denmark; 4 Institute of Clinical Research, University of Southern Denmark, Odense, Denmark; 5 SA MRC Developmental Pathways for Health Research Unit, Faculty of Health Sciences, University of the Witwatersrand, Johannesburg, 2193 South Africa

**Keywords:** Pre-conception, Weight loss, Obesity, Health behaviors, Psychosocial factors, Relationship dynamics, COM-B model, Weight stigma

## Abstract

**Background:**

The prevalence of overweight or obesity in women of reproductive age continues to increase. A high pre-pregnancy body mass index (BMI) has been shown to increase the risk of pregnancy complications and predispose offspring to childhood obesity. However, little is known about factors affecting women’s ability to achieve sustainable weight management and very few studies have applied behavior change theory to qualitative data.

**Aim:**

This study aimed to explore barriers and facilitators for weight management among women with overweight or obesity, who wanted to lose weight before pregnancy.

**Methods:**

We conducted semi-structured interviews with 17 women with a BMI ≥ 27 kg/m^2^, who planned to become pregnant in the near future. Data were analyzed using an abductive approach and the Capability, Opportunity, Motivation, and Behavior model was applied as a conceptual framework.

**Results:**

The women’s strongest motivator for pre-conception weight loss was their ability to become pregnant. Barriers to successful weight management included their partners’ unhealthy behaviors, mental health challenges, competing priorities, and internalized weight stigmatization. The women described careful planning, partners’ health behaviors, social support, and good mental health as facilitators for sustainable weight management.

**Conclusion:**

Our study provides insights into factors affecting weight management among women with overweight or obesity in the pre-conception period. Future interventions on weight management require a holistic approach, including a focus on social support, especially from the partner, and mental health, as well as an effort to limit internalized weight stigma.

## Background

The prevalence of overweight and obesity in women of reproductive age continues to increase [1]. Today, almost 40% of Danish pregnant women have pre-pregnancy overweight or obesity [2]. Overweight and obesity increase the risk of sub-fecundity (prolonged time to obtain pregnancy), infertility due to hormonal disturbances [3], and pregnancy complications, including early pregnancy loss, gestational diabetes mellitus (GDM), and preeclampsia [4–6]. In addition, offspring exposed to maternal overweight or obesity in utero have a 2–threefold higher risk of developing childhood obesity and an increased risk of developing type 2-diabetes compared to offspring of women with body mass index (BMI) < 25 kg/m^2^ [7–10]. The pre-conception phase refers to the time period before conception of a fetus. The World Health Organization has identified this period as one of the most important time points for health and disease development during the life-course, as it provides an opportunity to improve health, not only for women before, during, and after pregnancy, but also for the whole family, including the partner and the next generations [11, 12]. Despite the importance of weight reduction for women with overweight or obesity before they attempt to obtain pregnancy, very few interventions in the pre-conception period have specifically sought to achieve this [13]. Instead, interventions have focused on other health-related behaviors, such as smoking or folic acid supplementation [14, 15]. Consequently, there is limited evidence for successful weight management interventions targeting women of reproductive age [16, 17]. Existing evidence on barriers and facilitators for improving weight status among women in the pre-conception phase is scarce. Four qualitative studies, three from Australia and one from the UK including women with overweight or obesity who were in the pre-conception phase or pregnant have been identified [18–21]. All four studies found a lack of dietary knowledge, competing priorities, and the cost of healthy living as important barriers to achieving and sustaining a weight loss. Furthermore, Scott et al*.* and Kretowicz et al. also described family needs and low levels of partner support as additional barriers [18, 21]. Furthermore, studies suggest that some women with overweight or obesity do not perceive themselves at high risk of weight related pregnancy complications and may therefore lack motivation for behavior change to improve their weight [19, 22]. In addition, only one qualitative pre-conception study has applied behavior change theory (BCT) as a means for developing an intervention to achieve sustainable weight loss [18]. BCT has been suggested as an important tool to develop successful and more effective interventions by linking relevant causal factors of a behavior to appropriate change [23, 24]. We conducted a study exploring the barriers and facilitators for weight management among Danish women with overweight or obesity, who wanted to lose weight before pregnancy. We used the Behavior Change Wheel (BCW) by applying the Capability, Opportunity, Motivation, and Behavior (COM-B) model to categorize our data to gain an in-depth understanding of factors affecting weight management promoting behaviors in an individual, environmental, and social context [23].

## Method

### Study design and participants

This qualitative study applied semi-structured interviews with Danish women with a BMI ≥ 27 kg/m^2^, who wanted to lose weight and wanted to become pregnant in the nearest future. All participants were recruited through online platforms: i) a podcast and associated Instagram account called “The waiting room” focused on a variety of topics related to women wanting to become pregnant; ii) an online newsletter published by a commercial website called “Babyplan.dk” selling pregnancy test kits, ovulation test, and pregnancy vitamins; iii) websites used for recruitment of participants to research projects. Women, who wanted to participate, filled out an online form with their contact information and were subsequently contacted by phone. Women were eligible if they were actively trying to become pregnant or were planning to become pregnant within the next two years, wanted to lose weight before pregnancy, were between 18 and 34 years of age, and had a BMI ≥ 27 kg/m^2^. Exclusion criteria were diabetes and not being able to communicate in Danish. Participants were purposively selected to ensure maximum variety of socio-demographic and reproductive status.

Thirty-three women expressed interest in participating, and a total of 20 women were eligible out of whom 17 were interviewed. Three women dropped out before the interviews due to mental health challenges.

### Data collection

Data were collected from September to November 2021. The interview guide was developed, and pilot tested before data collection was initiated. The guide contained open-ended questions and covered aspects of overweight and pregnancy, food and eating habits, physical activity, and partner- and family influence on health-related behaviors. The interviews were conducted by MLP, a Danish female researcher with a nutritional background and similar age as the women. Six interviews were conducted face-to-face (in the participant’s own home or in a public library), and 11 interviews were performed virtually. All interviews were audio-recorded and transcribed verbatim using pseudonyms to maintain anonymity of participants. The interviews had an average duration of 81 min (range 50–111 min) and data collection continued until data saturation was reached. Transcripts were not returned to participants for comments or corrections.

### Data analysis

All interviews were analyzed using qualitative content analysis [25] and a step-wise, abductive approach [25, 26]. The COM-B model was used to categorize data according to facilitators and barriers in the context of both internal and external factors. The COM-B model consists of the three superordinate themes, capability (C), opportunity (O), and motivation (M) each having two subordinated frameworks. Capability includes ‘physical’ and ‘psychological’, opportunity includes ‘physiological’ and ‘social’, and motivation includes ‘automatic’ and ‘reflective’ [23, 27]. In this analysis the COM-B model was used for the identification of sub-themes and interpretation of patterns across the data [23]. The interview transcripts were initially analyzed by the first author and consulted with JAH. All identified themes, sub-themes, and concepts were reviewed, revised, and discussed within the research group to ensure consistency and agreement with the final analysis. MLP who is fluent in English translated the quotes from Danish to English herself. If there were any doubts about the translation, she consulted with the co-authors. Participants did not provide feedback on the analytical findings. NVivo version 12 software (QSR International Pty. Melbourne, Australia) was used to assist the coding. Interviews and analysis were performed with adherence to best-practice guidelines for qualitative research [28].

### Research ethics

All participants received written and verbal information about the study and gave written consent. They were guaranteed anonymity and informed that they could withdraw from the study at any time, without explanation. Participants were also informed that there were no right or wrong answers. In all phases of the project, we carefully considered language and terminologies trying to avoid stigmatizing and judgmental phrases that could inflict emotional damage. Ethical approval for qualitative research is not required in Denmark.

## Results

### Socio-demographic data

Table 1 describes the demographic characteristic of the study participants.

**Table 1:** Socio-demographic characteristics of study participants

Socio-demographics and characteristics	*N* = 17
**Age (years)**	28 (21–29)
**Body weight (kg)**	101.4 (20.5)
**BMI (kg/m**^**2**^**)**	32.7 [range 27.7–50.4]
**Relationship status**	
Married	3 (18%)
Not married, but living with a partner	11 (64%)
In a relationship, not living with the partner	3 (18%)
**Education**	
Primary school (9 years)	1 (6%)
Vocational education level	7 (41%)
Bachelor’s degree or equivalent	6 (35%)
Master’s degree or equivalent	3 (18%)
**Occupation**	
Work – full time	9 (53%)
Student – full time	7 (41%)
Maternity leave	1 (6%)
**Reproductive status**	
Have children	5 (29%)
**Miscarriages**	
Have had one or more miscarries	8 (47%)

Demographic data are presented as mean ± standard deviation (SD) for normally distributed variables, medians (interquartile ranges) for skewed variables, [range] or number (%). Body Mass Index (BMI)

### Themes according to the COM-B model

Key themes related to barriers and facilitators for weight management behaviors were identified by using the three superordinate themes of the COM-B model: capability, opportunity, and motivation, with the six additional subordinated frameworks and 18 sub-themes with associated concepts (Table 2).

**Table 2:** Coding framework based on key terms from the COM-B model: subthemes and concepts

COM-B	Framework	Subtheme	Concepts
**Capability**	Psychological	Action planning, control, and decision processes Knowledge Skills Practices	Continuity in behavioral change, “all or nothing” mentality Knowledge or misinformed perception of nutrition and physical activity about weight loss Skills to prepare and incorporate healthy food in everyday life, perceived competencies Eating practices and physical activity proclivity
	Physical	Bodily restriction	Excessive body weight, fatigued, restricted by limb pain or medical conditions to engage in physical activity
**Opportunity**	Social	Partner’s behavior Relationship dynamics Descriptive norms Social support	Partner’s eating preferences and willingness to behavior change, partner’s physical activity level, and physical fitness Health- or obesogenic promoting relationship, practical weight loss support, perception of how to be a good girlfriend Eating culture Perceived weight loss support from partner’s, friends, family, and co-workers
	Physical	Environment Competing priorities	Food accessibility at home, distance to fitness facilities, the weather Time restriction, children and/or partner’s first, work, studies, and duties
**Motivation**	Reflective	Beliefs about consequences Goals Intentions	Perceived risk of weight-related pregnancy complications Goal setting, positive reinforcement (ability to become pregnant, being eligible for fertility treatment, reduced risk of pregnancy complications, “a beautiful pregnant woman”) Want to be a good example for children
	Automatic	Stigmatization Identity Emotion Disengaged	Weight stigmatization, experienced, perceived, anticipated, and internalized “I have always been “the fat girl” in school”, “I am not the type of person that runs”, and “I am an emotional eater”. Self-acceptance vs. wanting to lose weight Stress, guilt, shame, self-blame, loneliness, internalized stigma, good mental health, optimism, hopes, and dreams of motherhood Weight denial, preferences for energy-dense food, and low levels of physical activity

#### Psychological capability

Sub-themes identified in psychological capability were *action planning, control, decision processes, knowledge, skills,* and *practices*. They reflect psychological aspects of the women’s ability to plan and have control over weight loss and maintenance, and how knowledge, skills, and practices acted as either facilitators or barriers. Most of the women had continuously fluctuated between weight loss- and weight gain periods throughout most of their teenage and adult life.

Sometimes, I get this “whatever… “it is all the same”” and then I eat whatever I want. But then I feel really guilty about it and then, we start all over again [with a weight loss] tomorrow.

(woman no. 5, 25 years, no children).

Women, who had succeeded in their planned behavior change and managed their eating, described structure and the ability to follow a plan as the main components for successful weight loss and long-term weight maintenance. In addition, lack of knowledge on appropriate nutrition in relation to weight loss was a consistent barrier among the women.

[When I am on a diet] it is always the same boring chicken, rice, and vegetables. When I think of healthy food, these are the first things that comes to my mind. It is chicken, cauliflower, broccoli, and that is just really boring.

(woman no. 4, 26 years, no children).

Some women pointed out lack of skills to incorporate healthy foods into their everyday life as a barrier to healthy eating.

It is definitely my lack of skills to prepare it [healthy foods] well enough and simply not be creative enough when it comes to implementing vegetables in the diet and everyday life.

(woman no. 9, 33 years, two children).

#### Physical capability

Within physical capability, we identified one sub-theme: *bodily restriction*. Some women felt physically restricted to be physically active (e.g., biking, running, fitness) and saw this as a major barrier for them to achieve weight loss. The physical restrictions were perceived to be caused by excessive body weight, fatigue, joint or limb pain, breathing difficulty, or other medical conditions.

*I cannot run, I cannot bike… I feel like there is an elephant on my chest when I go bicycling.*

(woman no. 16, 25 years, no children).

#### Social opportunities

Sub-themes within social opportunities included *the partner’s behavior, relationship dynamics, norms,* and *social support*, as well as how the external social surroundings affected the women’s behavior and their ability to reduce and maintain weight. The partners and their eating behaviors played a major role as either a facilitator or barrier, irrespectively of whether they were living together or not.

It is really difficult to have a healthy lifestyle when those you are close to [my boyfriend] has an unhealthy lifestyle.

(woman no. 5, 25 years, no children).

If the partner wanted to change diet and eat the same food as the woman while she was trying to lose weight this was a facilitator.

He tries to support me the best he can, so he also eats it [healthy foods for dinner] because he knows it is a support for me. He helps me.

(woman no. 13, 29 years, no children).

Grocery shopping, cooking and healthy eating within the household were for some women perceived solely as their responsibility with no social or practical support from the partners, and this proved to be a challenge for long-term sustainability of behavior changes.

I must take the lead. I am the only one who cooks dinner, and I make the grocery shopping list. I must keep pushing forward because he does not do it himself. He will join [the healthy eating] but he would not bother doing the work it requires himself.

(woman no. 17, 27 years, one child).

However, the women also reported not wanting their partners to be involved in a weight loss project together with them. This was rooted in weight loss being perceived as the women’s personal project. The women suspected their partners would lack motivation for long-term sustainability of healthy dietary and physical activity behaviors, and therefore be a barrier by double burdening the women with the responsibility for their own and their partners’ motivation and weight loss.

The relationship dynamics within the couple was also a factor noted by the women. For instance, some described quality time with their partner as often involving an energy-dense dinner or snacking together in the evening, which was perceived as a way to nurture the relationship. Declining or suggesting dietary alternatives were, therefore, challenging for the women, especially when the partner disagreed and kept “tempting” the woman by continuing eating energy dense foods.

We ate ice cream the other night. It was not because we were hungry, but because it was nice and ‘comfy’, and it is important that we have a good time together and that is our excuse to eat unhealthily. It is about having a good time together.

(woman no. 11, 28 years, no children).

Women, who perceived their partners as a support for engaging in physical activity, experienced a feeling of security and the support also facilitated the behavior long term.

My boyfriend has gone with me when I have done strength training and offered me support. He has shown me how to use the [fitness] machines.

(woman no. 10, 24 years, one child).

Many women described how healthy eating was not perceived as the norm among family and friends. This made it stressful and challenging for them to eat healthy when attending family dinners and social events as it was not perceived possible to eat differently than their peers. Consequently, some women reported feeling socially excluded when trying to change their dietary behavior.

When someone invites me to their birthday, the first thing that comes to my mind is: “oh no, I cannot go because I will have to eat something unhealthy, I will have to drink alcohol”, I do not want to do that. I cannot go.

(woman no. 4, 26 years, no children)

The majority of the women kept their weight loss plans a secret, as they had little faith in their peers’ understanding how challenging it was for them to lose weight. Some women did not want any “good advice” on weight loss, others would not risk being judged and feeling like a failure if they were unsuccessful in losing weight.

I might be afraid that if I say something [about my weight loss plans], and I fail [in losing weight], they will think “oh she really does not have any willpower”.

(woman no. 17, 27 years, one child).

#### Physical opportunity

Sub-themes identified in physical opportunity were *environment* and *competing priorities*. Physical opportunity describes external factors outside the women’s control. One was food availability at home, where the partners again played an important role in which food items were available in the household. The other environmental factor included poor access to fitness facilities, especially in rural areas where a fitness center could be many kilometers away or was too small to offer fitness classes.

The fitness center is far from here and that plays a role. It requires a lot of time [to get there] and it therefore becomes unmanageable for me [to go].

(woman no. 11, 28 years, no children).

The sub-theme of competing priorities was a re-occurring topic and barrier for almost all women interviewed. It was typically referred to as lack of time. Competing priorities included everyday tasks such as work, studies, the partners’ needs, household duties, or childcare.

I have shifting working hours. And when you have a two-year-old boy that you want to spend time with and everything that goes with having a child- doing laundry, tidying up, and cleaning—time just disappears so fast. For me, that is a big factor for why it is so challenging [to lose weight].

(woman no. 17, 27 years, one child).

#### Reflective motivation

Reflective motivation is related to self-conscious intentions and beliefs abouts what is good and bad [29]. Sub-themes identified within reflective motivation were *beliefs about consequences, goals,* and *intentions*. A predominant motivational theme for weight loss was fertility. The women believed that their body weight could affect their ability to become pregnant and that weight loss was a tangible method to improve the chances of pregnancy.

My goal with weight loss is not necessarily to look good or about the number of kilos I lose. It is because I want to become pregnant.

(woman no. 14, 21 years, no children).

For about half of the women interviewed, weight loss in relation to fertility treatment was a theme. In Denmark, fertility treatment is covered by the public health care system with the couple’s first child. However, a woman must have a BMI ≤ 30 kg/m^2^ to be eligible to treatment if she is under 35 years old [30].

There is a BMI cut-off of 30, right? And my BMI exceeds that. So, I am like “oh well, I will just have to lose a little weight” and that is what I am struggling with now… It’s really not that easy [to lose weight] and especially not when you have to.

(woman no. 11, 28 years, no children). Most women knew that an excessive body weight increases the risk of developing GDM and other unspecified, weight-related pregnancy complications. However, only a few women perceived themselves as being at high risk. Moreover, a few women were concerned about their appearance while being pregnant. These women believed that a weight loss would give them a visually attractive pregnancy, including the iconic round belly.

I want to lose weight before [pregnancy]. Both because I want to look prettier when I become pregnant and so that it will be possible to see. You know, I want to be a ‘pretty pregnant’, because I have seen some [women] where it was not possible to see that they were pregnant before they were rather far in their pregnancy ……., I do not want that.

(woman no. 16, 25 years, no children).

Finally, women, who already had children, were motivated as they wanted to give their children a better life without the same weight challenges as they themselves had experienced in their childhood.

We [my husband and I] talked a lot about how it will affect my son and our [future] children’s health. …….. it means a lot to me that our children do not have to go through the same as me.

(woman no. 7, 26 years, one child).

#### Automatic motivation

Automatic motivational factors are related to emotions and impulses that the individual is not in control of. These factors are directly associated with behavior and are proposed to mediate the associations between capability, opportunity, and the desired behavior [29].

Sub-themes identified in automatic motivation were *stigmatization, identity, emotion,* and *disengagement*. A predominant sub-theme was that the women had experienced weight related stigma from colleagues, classmates, partners, and family members. In the family setting, some women had experienced an ongoing focus on their body weight and weight loss throughout their childhood, adolescence, and adult life, especially from the women’s mothers.

All my life, my mom has been like this “I think you have gained some weight” or “now you lost a little weight” or “you look like this”.

(woman no. 8, 32 years, no children).

Internalized stigma was an underlining theme for many of the women. The women felt shameful and guilty about their bodies and were blaming themselves for repeatedly failing to lose weight or maintain the weight loss.

I have been really sad about it [not being able to lose weight] many times. I have been feeling useless. I have had a really, really low self-esteem, because I have felt guilt. Guilty that I could not make it [the weight loss] work, that I could not maintain it [the weight loss], that I could not get started, and that I could not keep going.

(woman no. 7, 26 years, one child).

The vast majority of the women described themselves as having distinct identities related to their health or weight status which may present as a barrier to engaging in weight-loss-promoting behaviors.

I have always been the fat girl, ever since kindergarten.

(woman no. 17, 27 years, one child).

Several of the interviewed women felt torn between an identity of wanting to be a member of the ‘body positivity movement’ while wanting to lose weight. Some practiced and promoted pride in their own body image, for example on social media or within their social networks, while simultaneously secretly wanting to lose weight in relation to the desire of becoming pregnant. The women described how these two aspects were contradictory.

I try to be body-positive and, like, try to be proud of myself and how I look. That does not go very well together with”okay, I am happy with my mommy-tummy, but I would like to lose weight”. That is two things that apparently are very conflicting with each other in our society. You cannot be happy with your self-image and at the same time have a desire to change it.

(woman no. 17, 27 years, one child).

Another prominent sub-theme was the women’s emotional responses to their body weight and weight loss attempts and how this affected their mental health and ability to lose weight in an ongoing vicious circle.

Very often, my mental health is a factor [for weight loss]. If I am out of balance, I end up eating takeaway or something like that.

(woman no. 5, 26 years, no children).

Furthermore, being ineligible for fertility treatment because of a too high BMI affected women’s mental health very negatively, and this was as a major barrier to weight loss among those women.

Everything suggests that I am stressed, because I feel guilty every time I eat, or every time I do not exercise and stuff like that because I know I have to lose weight in order for us to enroll in [fertility-]treatment. ………. I mean, I would love to [lose weight], and I try, but I do not really try. It is the same every day. It is just really hard.

(woman no. 4, 26 years, no children).

## Discussion

### Key findings

This study examined facilitators and barriers for engaging in weight loss and maintenance promoting behaviors before pregnancy among Danish women living with overweight or obesity. To our knowledge this is the first study to explore the factors affecting weight management in the pre-conception period using the framework of the COM-B model. The model was used to identify to identify the main components relevant for interventions aiming to obtain and support sustainable behavior change before pregnancy.

Our results suggest that to achieve sustainable behavior change for weight management in the pre-conception phase, several key components within the COM-B framework are important. All the interviewed women spoke of their ongoing struggle to lose weight, and most described this as being a challenge ever since they were teenagers. The women had experienced weight variations and unexplained weight gain. These results support the work of other studies investigating experiences with weight loss among women living with overweight or obesity [31–34].

The importance of the women’s partners was a key element within the social opportunity theme in the COM-B model. The women described challenges with the partner’s attitudes and behaviors in relation to eating, physical activity and lack of support to their weight loss plans, although in some cases, the partners could also play a supportive role. These findings are in harmony with existing qualitative work in this area. Previous research by Scott et al*.* performed in Australia [18] and Kretowicz et al*.* from the UK [21] also highlighted the partners as a barrier to eating more healthy because they prepared or ate unhealthy food in front of the women or were not willing to undergo dietary changes themselves [18, 21, 32]. Similar issues on the partner as an obstacle for weight loss were identified in a Swedish study, where the partners tempted the women with unhealthy food, brought it to the households, or insisted the women should cook their traditional food, resulting in the women cooking two separate dinners [32]. In accordance with our findings, these studies found that the partner also could act as an enabler for weight loss if the weight loss became a shared goal, if the partner accommodated the dietary- and schedule changes, and provided practical support (e.g. childcare). Our analysis also shows that couple dynamics can have a strong influence on the women’s ability to sustain behavior change. Thus, it is essential that weight loss interventions address the complexity of losing weight in this larger social context [18].

The women’s mental health and emotional responses to their body weight and desired pregnancy were important facets of automatic motivation to adopt and sustain weight loss promoting behaviors. Unprompted, most women reported that their mental health (e.g., feeling blue, stressed, or depressed with low mental energy) was a barrier for weight loss because those feelings limited their motivation for preparing and eating healthy foods, being physically active, or both. In line with this finding, mental health challenges have been recognized as a weight loss barrier for women in the reproductive age in several other studies [18, 31, 34]. In addition, the women in our study characterized themselves with negative identities and stereotypes and were blaming themselves for their excessive body weight and inability to lose weight, suggesting that most had internalized weight stigma. It is well documented that women living with overweight or obesity in high-income societies experience weight stigma from educators, colleagues, the media, friends, partners and family members [35–38]. The blame for an excessive body weight is placed upon the women, which can lead to internalization of the stigma with negative consequences for self-perception and identity [39–42]. Individuals with internalized weight stigma are at increased risk of having a low self-esteem, feeling lonely and experiencing depression and anxiety [43–45]—factors found to be associated with unsuccessful weight loss and maintenance [44, 46–48], which was also the case for women in our study. Seen from the perspective of the COM-B framework, our data suggest that improvement of mental health and reduction of internalized weight stigma would improve motivational aspects by addressing the capability and opportunity for engaging in sustainable behavior change to achieve and maintain weight loss.

Most women were familiar with some weight-related pregnancy complications, especially GDM, but this was not a motivational factor for weight loss, as they did not perceive themselves at higher risk. Why the women did not perceive them self at risk is unknown. It could hypothetically be because the women interviewed were young and most of them did not have any challenges with their health. It may therefore be too abstract for the women to believe they are at real risk. Very few were knowledgeable of the potential risk to their future child, and it was therefore not a motivational factor for weight loss. Rather, the predominant motivation for weight loss was enhancing chances of conception. This is contrary to Scott and colleagues, who found that motivation was driven by wanting a better physical and mental health for the women’s family. This difference might be explained by the fact that all women in Scott et al*.* already had children, and only two women intended pregnancy within the next 12 months [18]. In contrast, all our participants wanted to become pregnant in the nearest future, and 2/3 were nulliparous. This is an important finding, as the difference suggests that motivations for weight loss might differ between parous and nulliparous women. Potential motivational differences for weight loss between parous and nulliparous women in the pre-conception period needs to be investigated further to tailor interventions to fit the motivational factors of this specific target group.

Most of the women in our study could describe the national Danish recommendations for healthy eating in great detail. The finding is opposing to many other studies where an insufficient level of nutritional knowledge has been identified as barrier for weight loss and healthy eating [18, 20, 21]. However, few of the women in our study translated the knowledge into practice due to the components identified in social opportunity, and automatic- and reflective motivation. This underlines that provision of knowledge cannot stand alone in behavior change interventions. This aspect is well described in several behaviors change models, including the COM-B model [23].

### Implications for practice and research

The current study provides essential insights of importance for interventions aiming to obtain and sustain weight loss in women with overweight or obesity who want to lose weight in the pre-conception period. It is clear from the results that a successful intervention must tackle wider contextual factors of the woman’s social and physical environment, and not just focus on factors operating at the individual level. Furthermore, interventions should include a focus on the women’s mental health and self-esteem, self-efficacy, and self-acceptance. In addition, we suggest a more holistic family approach, as the couples’ or families’ needs, and norms have been shown to be critical to sustain behavior change [18, 21, 32]. More specifically, future interventions should consider the impact of the partners and ideally include them as part of a family intervention to enhance long-term sustainability of healthy behaviors. To address this issue, further qualitative research is needed on the partners’ perceptions and motivations for engaging in weight loss and maintenance promoting behaviors. Likewise, there is a need for similar research within various other socio-cultural settings.

### Strengths and limitations

This study has several strengths. The COM-B model is an established method used extensively in behavior change interventions, including dietary and physical activity interventions [27, 49, 50]. We used the model systematically to explore the broader and in depth social and environmental perspectives for weight management, which we are the first to do for this target group. In addition, our study is the first to interview women that all wanted to become pregnant in the nearest future, and it thus provides previously unexplored insights to motivations in the pre-conception period. Also, the women displayed a diverse profile across socio-economic characteristics, living area (rural and urban), BMI, age, family circumstances and included both nulliparous and parous women.

However, our study is not without some limitations. 15 out of the 17 women were recruited from two online platforms targeting women, who want to become pregnant. The interviewed women may be more interested in and informed about health in pregnancy and weight loss as they had sought the online platforms. Moreover, the women were not culturally diverse. The cultural implications of weight loss-promoting behaviors need to be considered and should be explored in future research to ensure culturally compatible interventions. Consequently, the barriers and facilitators identified and described in our study may not be representative of the entire general population of women with overweigh or obesity, who want to lose weight prior to a pregnancy, in Denmark. Also, we did not include interviews with partners or health care professionals. Such triangulation of data sources might shed additional light on the factors affecting behaviors relevant for weight loss and maintenance. Nevertheless, we find that focusing on the women’s experiences in the first instance generated valuable insights about how to frame future interventions. Lastly, MLP was mainly involved in the coding, and even though she closely consulted all findings within the research group, this may have increased the possibility of missing themes.

## Conclusion

The pre-conception period is considered one of the most important phases during the life-course to prevent obesity and improve health, not only for the women themselves but also for the next generations. However, achieving, and sustaining behavior change is a complex and challenging task influenced by multiple social, environmental, and behavioral factors. This study provides new insights into factors of importance for obtaining and sustaining behavior change among Danish women with overweight and obesity who want to lose weight before pregnancy. By applying the COM-B framework, this study identified the complexities inherent in intervention development at individual, social, and environmental levels. Promotion of behavior change for this group of women requires a holistic approach that strengthens the components of capability, opportunity, and motivation. Important considerations for future interventions include social support, especially from the partner, improving mental health, and limiting internalized weight stigma.

## Data Availability

The datasets generated and analyzed during the current study are not publicly available as the participants did not give their informed consent to share their responses public but are available from the corresponding author on reasonable request.

## Abbreviations

BMI: Body mass index
BCT: Behavior change theory
BCW: Behavior Change Wheel
COM-B: Capability, Opportunity, Motivation, and Behavior model
GDM: Gestational diabetes mellitus

## References

[1] Dağ ZÖ, Dilbaz B (2015). Impact of obesity on infertility in women. J Turkish Ger Gynecol Assoc..

[2] Nyfødte og fødsler (2021). esundhed.dk. 2022 [cited 2022 Sep 27]. Available from: https://www.esundhed.dk/Emner/Graviditet-foedsler-og-boern/Nyfoedte-og-foedsler-1997-#tabpanel6B95F8298EB444F48C3403E7B75B7202

[3] World Health Organization. ICD-11 for Mortality and Morbidity Statistics. Geneva, 2018. 2020. 2020 [cited 2022 May 24]. p. https://icd.who.int/browse11/l-m/en. Available from: https://icd.who.int/browse11/l-m/en#/http%3A%2F%2Fid.who.int%2Ficd%2Fentity%2F30659757

[4] Poston L, Caleyachetty R, Cnattingius S, Corvalán C, Uauy R, Herring S, et al. Preconceptional and maternal obesity: epidemiology and health consequences. lancet Diabetes Endocrinol. 2016;4(12):1025–36. Available from: https://pubmed.ncbi.nlm.nih.gov/27743975/10.1016/S2213-8587(16)30217-0 27743975

[5] Stubert J, Reister F, Hartmann S, Janni W (2018). The Risks Associated With Obesity in Pregnancy. Dtsch Arztebl Int..

[6] D’Souza R, Horyn I, Pavalagantharajah S, Zaffar N, Jacob CE. Maternal body mass index and pregnancy outcomes: a systematic review and metaanalysis. Am J Obstet Gynecol MFM [Internet]. 2019;1(4). Available from: https://pubmed.ncbi.nlm.nih.gov/33345836/10.1016/j.ajogmf.2019.100041 33345836

[7] Adane AA, Dobson A, Tooth L, Mishra GD. Maternal preconception weight trajectories are associated with offsprings’ childhood obesity. Int J Obes (Lond). 2018;42(7):1265–74. Available from: https://pubmed.ncbi.nlm.nih.gov/29795458/10.1038/s41366-018-0078-1 29795458

[8] Gaillard R, Steegers EAP, Duijts L, Felix JF, Hofman A, Franco OH, et al. Childhood cardiometabolic outcomes of maternal obesity during pregnancy: the Generation R Study. Hypertens (Dallas, Tex 1979). 2014;63(4):683–91. Available from: https://pubmed.ncbi.nlm.nih.gov/24379180/10.1161/HYPERTENSIONAHA.113.02671 24379180

[9] Yu Z, Han S, Zhu J, Sun X, Ji C, Guo X. Pre-pregnancy body mass index in relation to infant birth weight and offspring overweight/obesity: a systematic review and meta-analysis. PLoS One. 2013;8(4). Available from: https://pubmed.ncbi.nlm.nih.gov/23613888/10.1371/journal.pone.0061627 PMC3628788 23613888

[10] Lahti-Pulkkinen M, Bhattacharya S, Wild SH, Lindsay RS, Räikkönen K, Norman JE, et al. Consequences of being overweight or obese during pregnancy on diabetes in the offspring: a record linkage study in Aberdeen, Scotland. Diabetologia. 2019;62(8):1412–9. Available from: https://link.springer.com/article/10.1007/s00125-019-4891-4 10.1007/s00125-019-4891-4 PMC6647186 31214738

[11] World Health Organisation. Report of the commission on ending childhood obesity. WHO, Geneva, Switzerland, 2016. [cited 2022 Apr 1]. Available from: https://www.who.int/publications/i/item/9789241510066

[12] KM R, AL Y. Weight Gain During Pregnancy: Reexamining the Guidelines. 2009 Dec 14 [cited 2022 Jul 7]; Available from: https://pubmed.ncbi.nlm.nih.gov/20669500/20669500

[13] Hanson M, Barker M, Dodd JM, Kumanyika S, Norris S, Steegers E, et al. Interventions to prevent maternal obesity before conception, during pregnancy, and post partum. lancet Diabetes Endocrinol. 2017;5(1):65–76. Available from: https://pubmed.ncbi.nlm.nih.gov/27743974/10.1016/S2213-8587(16)30108-5 27743974

[14] Temel S, Van Voorst SF, Jack BW, Denktaş S, Steegers EAP. Evidence-based preconceptional lifestyle interventions. Epidemiol Rev. 2014;36(1):19–30. Available from: https://pubmed.ncbi.nlm.nih.gov/23985430/10.1093/epirev/mxt003 23985430

[15] Poston L, Caleyachetty R, Cnattingius S, Corvalán C, Uauy R, Herring S, et al. Preconceptional and maternal obesity: epidemiology and health consequences. lancet Diabetes Endocrinol. 2016;4(12):1025–36. Available from: https://pubmed.ncbi.nlm.nih.gov/27743975/10.1016/S2213-8587(16)30217-0 27743975

[16] Hutchesson MJ, Hulst J, Collins CE. Weight Management Interventions Targeting Young Women: A Systematic Review. J Acad Nutr Diet. 2013;113(6):795–802. Available from: http://www.jandonline.org/article/S221226721300097X/fulltext 10.1016/j.jand.2013.01.015 23473986

[17] Opray N, Grivell RM, Deussen AR, Dodd JM. Directed preconception health programs and interventions for improving pregnancy outcomes for women who are overweight or obese. Cochrane database Syst Rev. 2015;2015(7). Available from: https://pubmed.ncbi.nlm.nih.gov/26171908/10.1002/14651858.CD010932.pub2 PMC10656571 26171908

[18] Scott J, Oxlad M, Dodd J, Szabo C, Deussen A, Turnbull D. Creating Healthy Change in the Preconception Period for Women with Overweight or Obesity: A Qualitative Study Using the Information-Motivation-Behavioural Skills Model. J Clin Med. 2020;9(10):1–20. Available from: https://pubmed.ncbi.nlm.nih.gov/33086583/10.3390/jcm9103351 PMC7603106 33086583

[19] Khan NN, Boyle J, Lang AY, Harrison CL. Preconception Health Attitudes and Behaviours of Women: A Qualitative Investigation. Nutrients. 2019;11(7). Available from: https://pubmed.ncbi.nlm.nih.gov/31261954/10.3390/nu11071490 PMC6682867 31261954

[20] Benton MR, Tape N, Deussen AR, Turnbull D, Dodd JM (2021). Barriers to and facilitators for addressing overweight and obesity before conception: A qualitative study. Women and Birth.

[21] Kretowicz H, Hundley V, Tsofliou F. Exploring the Perceived Barriers to Following a Mediterranean Style Diet in Childbearing Age: A Qualitative Study. Nutrients. 2018;10(11). Available from: https://pubmed.ncbi.nlm.nih.gov/30404231/10.3390/nu10111694 PMC6266554 30404231

[22] van der Zeea B, de Beaufort ID, Steegers EAP, Denktaş S. Perceptions of preconception counselling among women planning a pregnancy: a qualitative study. Fam Pract. 2013;30(3):341–6. Available from: https://pubmed.ncbi.nlm.nih.gov/23180815/10.1093/fampra/cms074 23180815

[23] Michie S, van Stralen MM, West R (2011). The behaviour change wheel: A new method for characterising and designing behaviour change interventions. Implement Sci..

[24] Bartholomew LK, Mullen PD. Five roles for using theory and evidence in the design and testing of behavior change interventions. J Public Health Dent. 2011;71 Suppl 1(SUPPL. 1). Available from: https://pubmed.ncbi.nlm.nih.gov/21656946/10.1111/j.1752-7325.2011.00223.x 21656946

[25] Schreier M. Qualitative Content Analysis in Practice. 1st ed. SAGE Publ.; 2012. p. 1–272.

[26] Graneheim UH, Lindgren BM, Lundman B. Methodological challenges in qualitative content analysis: A discussion paper. Nurse Educ Today. 2017;56:29–34. Available from: https://pubmed.ncbi.nlm.nih.gov/28651100/10.1016/j.nedt.2017.06.002 28651100

[27] Willmott TJ, Pang B, Rundle-Thiele S (2021). Capability, opportunity, and motivation: an across contexts empirical examination of the COM-B model. BMC Public Health.

[28] Tong A, Sainsbury P, Craig J. Consolidated criteria for reporting qualitative research (COREQ): a 32-item checklist for interviews and focus groups. Int J Qual Heal Care. 2007;19(6):349–57. Available from: https://academic.oup.com/intqhc/article/19/6/349/1791966 10.1093/intqhc/mzm042 17872937

[29] Willmott TJ, Pang B, Rundle-Thiele S. Capability, opportunity, and motivation: an across contexts empirical examination of the COM-B model. BMC Public Heal 2021 211 [Internet]. 2021 May 29 [cited 2022 Jul 15];21(1):1–17. Available from: https://bmcpublichealth.biomedcentral.com/articles/10.1186/s12889-021-11019-w 10.1186/s12889-021-11019-w PMC8164288 34051788

[30] Alsbjerg B, Petersen KB. Overvægt, fedme og fertilitetsbehandling. 2013. p. 1–19.

[31] Lim S, Smith CA, Costello MF, MacMillan F, Moran L, Ee C. Barriers and facilitators to weight management in overweight and obese women living in Australia with PCOS: A qualitative study. BMC Endocr Disord. 2019;19(1):1–9. Available from: https://bmcendocrdisord.biomedcentral.com/articles/10.1186/s12902-019-0434-8 10.1186/s12902-019-0434-8 PMC6813064 31647000

[32] Hammarström A, Wiklund AF, Lindahl B, Larsson C, Ahlgren C (2014). Experiences of barriers and facilitators to weight-loss in a diet intervention - a qualitative study of women in Northern Sweden. BMC Womens Health..

[33] Sharifi N, Mahdavi R, Ebrahimi-Mameghani M (2013). Perceived Barriers to Weight loss Programs for Overweight or Obese Women. Heal Promot Perspect..

[34] Quinn DM, Puhl RM, Reinka MA (2020). Trying again (and again): Weight cycling and depressive symptoms in U.S. adults. PLoS One..

[35] Greenberg BS, Eastin M, Hofschire L, Lachlan K, Brownell KD (2003). Portrayals of Overweight and Obese Individuals on Commercial Television. Am J Public Health..

[36] Puhl R, Brownell KD. Bias, discrimination, and obesity. Obes Res. 2001;9(12):788–805. Available from: https://pubmed.ncbi.nlm.nih.gov/11743063/10.1038/oby.2001.108 11743063

[37] Himes SM, Thompson JK. Fat stigmatization in television shows and movies: a content analysis. Obesity (Silver Spring). 2007;15(3):712–8. Available from: https://pubmed.ncbi.nlm.nih.gov/17372322/10.1038/oby.2007.635 17372322

[38] Brown A, Flint SW, Batterham RL. Pervasiveness, impact and implications of weight stigma. EClinicalMedicine. 2022;47. Available from: https://pubmed.ncbi.nlm.nih.gov/35497065/10.1016/j.eclinm.2022.101408 PMC9046114 35497065

[39] Puhl RM, Heuer CA (2010). Obesity stigma: Important considerations for public health. Am J Public Health..

[40] Olson KL, Landers JD, Thaxton TT, Emery CF. The pain of weight-related stigma among women with overweight or obesity. Stigma Heal. 2019;4(3):243–6. Available from: https://pubmed.ncbi.nlm.nih.gov/31592443/10.1037/sah0000137 PMC6779419 31592443

[41] Fulton M, Srinivasan VN. Obesity, Stigma And Discrimination. StatPearls. StatPearls Publishing; 2020 [cited 2022 May 29]. Available from: https://www.ncbi.nlm.nih.gov/books/NBK554571/32119458

[42] Williams O, Annandale E. Obesity, stigma and reflexive embodiment: Feeling the ‘weight’ of expectation. Heal (United Kingdom). 2020;24(4):421–41. Available from: https://journals.sagepub.com/doi/full/10.1177/1363459318812007?casa_token=qdqyUwU572EAAAAA%3AKVGmFS7U6e3tNFxxejhZzqhqXS0Foc33K-CLJ-YjVAozZ3FA7H4Jif60m6tVvLp0wavZ71g8MnU 10.1177/1363459318812007 30428717

[43] Emmer C, Bosnjak M, Mata J. The association between weight stigma and mental health: A meta-analysis. Obes Rev. 2020;21(1). Available from: https://pubmed.ncbi.nlm.nih.gov/31507062/10.1111/obr.12935 31507062

[44] Lee KM, Hunger JM, Tomiyama AJ. Weight stigma and health behaviors: evidence from the Eating in America Study. Int J Obes 2021 457. 2021;45(7):1499–509. Available from: https://www.nature.com/articles/s41366-021-00814-5 10.1038/s41366-021-00814-5 PMC8236399 33934109

[45] Puhl RM, Heuer CA. The stigma of obesity: a review and update. Obesity (Silver Spring). 2009;17(5):941–64. Available from: https://pubmed.ncbi.nlm.nih.gov/19165161/10.1038/oby.2008.636 19165161

[46] Puhl RM, Quinn DM, Weisz BM, Suh YJ. The Role of Stigma in Weight Loss Maintenance Among U.S. Adults. Ann Behav Med. 2017;51(5):754–63. Available from: https://pubmed.ncbi.nlm.nih.gov/28251579/10.1007/s12160-017-9898-9 28251579

[47] Meadows A, Higgs S (2019). Internalised weight stigma moderates the impact of a stigmatising prime on eating in the absence of hunger in higher- but not lower-weight individuals. Front Psychol.

[48] Bidstrup H, Brennan L, Kaufmann L, de la Piedad Garcia X. Internalised weight stigma as a mediator of the relationship between experienced/perceived weight stigma and biopsychosocial outcomes: a systematic review. Int J Obes 2021 461 [Internet]. 2021 Oct 9 [cited 2022 Apr 8];46(1):1–9. Available from: https://www.nature.com/articles/s41366-021-00982-4 10.1038/s41366-021-00982-4 PMC8501332 34628466

[49] Boyd J, McMillan B, Easton K, Delaney B, Mitchell C. Utility of the COM-B model in identifying facilitators and barriers to maintaining a healthy postnatal lifestyle following a diagnosis of gestational diabetes: a qualitative study. BMJ Open. 2020;10(8). Available from: https://pubmed.ncbi.nlm.nih.gov/32753450/10.1136/bmjopen-2020-037318 PMC7406111 32753450

[50] Howlett N, Schulz J, Trivedi D, Troop N, Chater A. A prospective study exploring the construct and predictive validity of the COM-B model for physical activity. J Health Psychol. 2019;24(10):1378–91. Available from: https://pubmed.ncbi.nlm.nih.gov/29172808/10.1177/1359105317739098 29172808

